# Impact of endoscopic submucosal dissection and epithelial cell sheet engraftment on systemic cytokine dynamics in patients with oesophageal cancer

**DOI:** 10.1038/s41598-021-94871-5

**Published:** 2021-07-27

**Authors:** Akira Yoshida, Tomoaki Takata, Tsutomu Kanda, Naoyuki Yamaguchi, Hitomi Minami, Kazuhiko Nakao, Shinichiro Kobayashi, Susumu Eguchi, Hajime Isomoto

**Affiliations:** 1 Division of Gastroenterology and Nephrology, Faculty of Medicine, Tottori University, Yonago, Tottori 683-8504 Japan; 2 Department of Gastroenterology and Hepatology, Nagasaki University Hospital, Nagasaki, 852‐8501 Japan; 3 Department of Surgery, Nagasaki University Graduate School of Biomedical Sciences, Nagasaki, 852‐8501 Japan

**Keywords:** Cancer, Gastroenterology

## Abstract

The tumour microenvironment (TME) plays an important role in cancer development, progression, and metastasis. Various cytokines are present in the TME in oesophageal cancer. Oesophageal stricture is a major complication of endoscopic submucosal dissection (ESD) for oesophageal cancer, and inflammatory cytokines are closely related to its pathogenesis. However, the cytokine crosstalk involved in the oesophageal cancer TME and post-ESD stricture has not been fully elucidated. This study investigated the comprehensive cytokine dynamics following ESD in patients with oesophageal cancer. In addition, the effect of a novel preventive technique for post-ESD stricture, autologous cell sheet engraftment, on cytokine levels was evaluated. Various pro-inflammatory and anti-tumorigenic cytokines were elevated in patients with oesophageal cancer, and ESD transiently influenced cytokine concentrations. IL-1β and TNF-α, two major pro-inflammatory cytokines that induce oesophageal stricture, were significantly suppressed by cell sheet engraftment. In conclusion, this study revealed the distinct cytokine dynamics after ESD in patients with oesophageal cancer, together with the effect of autologous cell sheet engraftment on cytokine fluctuation. These results can accelerate research on the TME and therapeutic strategies for oesophageal cancer.

## Introduction

The incidence of oesophageal cancer is gradually increasing worldwide. Oesophageal squamous cell carcinoma and oesophageal adenocarcinoma are the two major subtypes of oesophageal cancer, with oesophageal squamous cell carcinoma accounting for approximately 90% of the cases^1^. Smoking and alcohol consumption are the major risk factors for oesophageal squamous cell carcinoma, whereas gastrooesophageal reflux disease and obesity are risk factors for oesophageal adenocarcinoma. Chronic exposure to carcinogenic substrates induces inflammation, reactive oxygen species production, and DNA damage, leading to the development and progression of oesophageal cancer. Recent studies have demonstrated that the tumour microenvironment (TME) plays an essential role in the progression and metastasis of oesophageal cancer^2,3^. The TME comprises immune cells, fibroblasts, and the extracellular matrix. In particular, pro-inflammatory cytokines, chemokines, and the crosstalk between these are major regulators of the TME in oesophageal cancer. Therefore, it is important to understand the comprehensive role of these cytokine interactions in the TME for the treatment of oesophageal cancer.

Therapeutic approaches for oesophageal cancer have advanced over the past decade. Endoscopic submucosal dissection (ESD) is a major therapeutic option for oesophageal cancer^4^. ESD is an optimal procedure for *en bloc* resection of cancers limited to intraepithelial or lamina propria mucosae^5^. ESD was initially performed in superficial oesophageal neoplasms, and with advances in technologies, the application of ESD has shifted to larger lesions. However, because dissection of the oesophageal mucosa causes inflammation and ulceration, oesophageal stricture is a serious complication after aggressive ESD^6^. Several treatments have been suggested for the prevention of oesophageal strictures after ESD. Local injection and systemic administration of steroids are expected to prevent post-ESD strictures by suppressing inflammation^7^. Cell transplantation and autologous mucosal cell sheet engraftment were recently developed for the prevention of oesophageal strictures^5^. We recently demonstrated that tissue-engineered autologous oral mucosal epithelial cell sheets could be transplanted without significant complications after ESD^8^.

Considering the complex crosstalk among various cytokines during the progression of oesophageal cancer and the inflammatory response after ESD, the comprehensive role of these cytokines in patients with oesophageal cancer who have undergone ESD remains to be elucidated. In addition, reports describing cytokine dynamics after cell sheet transplantation are scarce. Therefore, in the present study, we aimed to investigate the changes in systemic cytokine levels in patients with oesophageal cancer, and evaluated the effect of cell sheet transplantation from this perspective.

## Results

### Patient characteristics

The characteristics of 28 patients (67.2 ± 7.6 years, 25 men and 3 women) are summarized in Table 1. There were no significant differences in age, sex, resection area, and post-ESD stricture between patients with or without cell sheet engraftment. Patients with cell sheets had a significantly larger resection circumstance (Table 1). The patients’ blood at baseline were measured for complete blood count, C-reactive protein level, neutrophil-to-lymphocyte ratio (NLR), and platelet-to-lymphocyte ratio (PLR). There were no significant differences among these laboratory results between the two groups (Table 2).

**Table 1:** Patient characteristics.

Cell sheet engraftment	Oesophageal cancer (n = 28)		p value
	−	+	
Number	18	10	
Age, years	68.6 ± 7.8	64.8 ± 7.1	NS
Sex (male/female)	16 / 2	9 / 1	NS
Resection area, cm^2^	46.3 ± 16.0	34.1 ± 20.4	NS
Circumstance of resection, %	73.6 ± 21.2	89.3 ± 6.3	< 0.05
**Site, n (%)**			
Ce	1 (5.6)	0	
Mt	6 (33.3)	8 (80.0)	
Lt	9 (50.0)	2 (20.0)	
Ae	2 (11.1)	0	
**Depth, n (%)**			
EP	8 (44.4)	1 (10.0)	
LPM	9 (50.0)	6 (60.0)	
MM	0	2 (20.0)	
SM	1 (5.6)	1 (10.0)	
**Post ESD stricture, n (%)**	2 (11.1)	4 (40.0)	NS

*ESD* endoscopic submucosal dissection, *Ce* cervical oesophagus, *Mt* middle thoracic oesophagus, *Lt* lower thoracic oesophagus, *Ae* abdominal oesophagus epithelium, *LPM* lamina propria mucosa, *MM* muscularis mucosae, *SM* submucosa.

**Table 2:** Laboratory results.

Cell sheet engraftment	Oesophageal cancer (n = 28)		p value
	−	+	
Number	18	10	
White blood cell count, 10^3^/μL	5.5 ± 1.3	5.1 ± 0.9	NS
Haemoglobin, g/dL	13.1 ± 1.6	14.0 ± 1.1	NS
Platelet, 10^6^/μL	1.9 ± 0.6	2.2 ± 0.3	NS
C-reactive protein	0.08 ± 0.17	0.08 ± 0.05	NS
NLR	2.11 ± 1.27	1.62 ± 0.60	NS
PLR	12.6 ± 4.9	12.8 ± 2.8	NS

*NLR* neutrophil to lymphocyte ratio, *PLR* platelet to lymphocyte ratio, *NS* not significant.

### Cytokines in control and patients with oesophageal cancer

**Figure 1:**
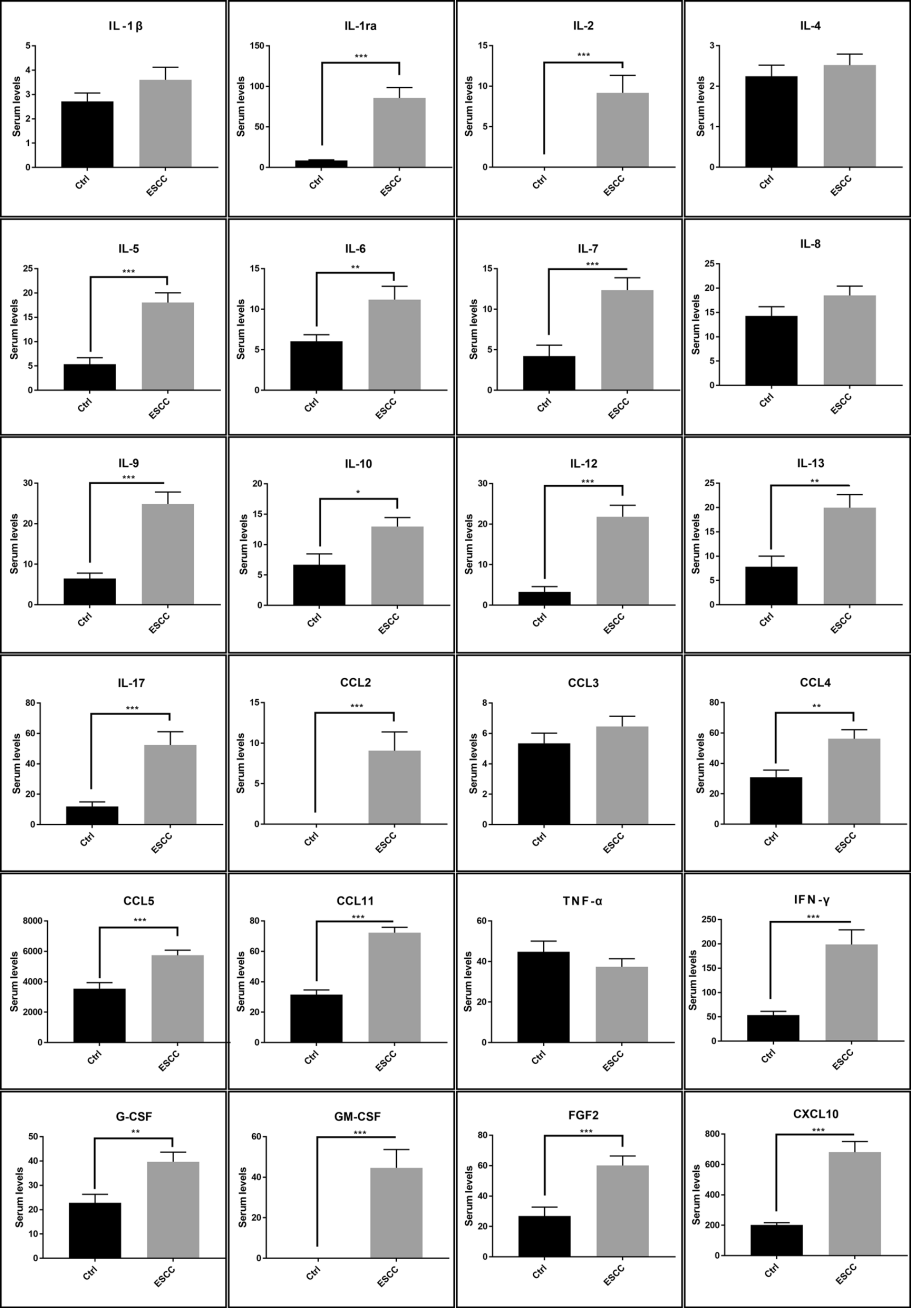
Comparison in systemic cytokines. Cytokines in healthy subjects and patients with oesophageal squamous cell carcinoma were compared. Bars indicate average ± SEM. *p < 0.05; **p < 0.01; ***p < 0.001.

To investigate the effect of oesophageal cancer on systemic cytokines, we compared cytokine levels between healthy subjects and patients with oesophageal squamous cell carcinoma. Patients with oesophageal cancer showed significantly higher levels of interleukin (IL)-1ra, IL-2, IL-5, IL-6, IL-7, IL-9, IL-10, IL-12, IL-13, IL-17, CC chemokine ligand (CCL)2, CCL4, CCL5, CCL11, interferon (IFN)-γ, granulocyte-colony stimulating factor (G-CSF), granulocyte–macrophage colony-stimulating factor (GM-CSF), fibroblast growth factor (FGF)2, and CXC chemokine ligand (CXCL)10. The levels of IL-2, CCL2, and GM-CSF were below the detection limit in healthy subjects (summarised in Fig. 1).

### Time course of systemic cytokines after ESD

**Figure 2:**
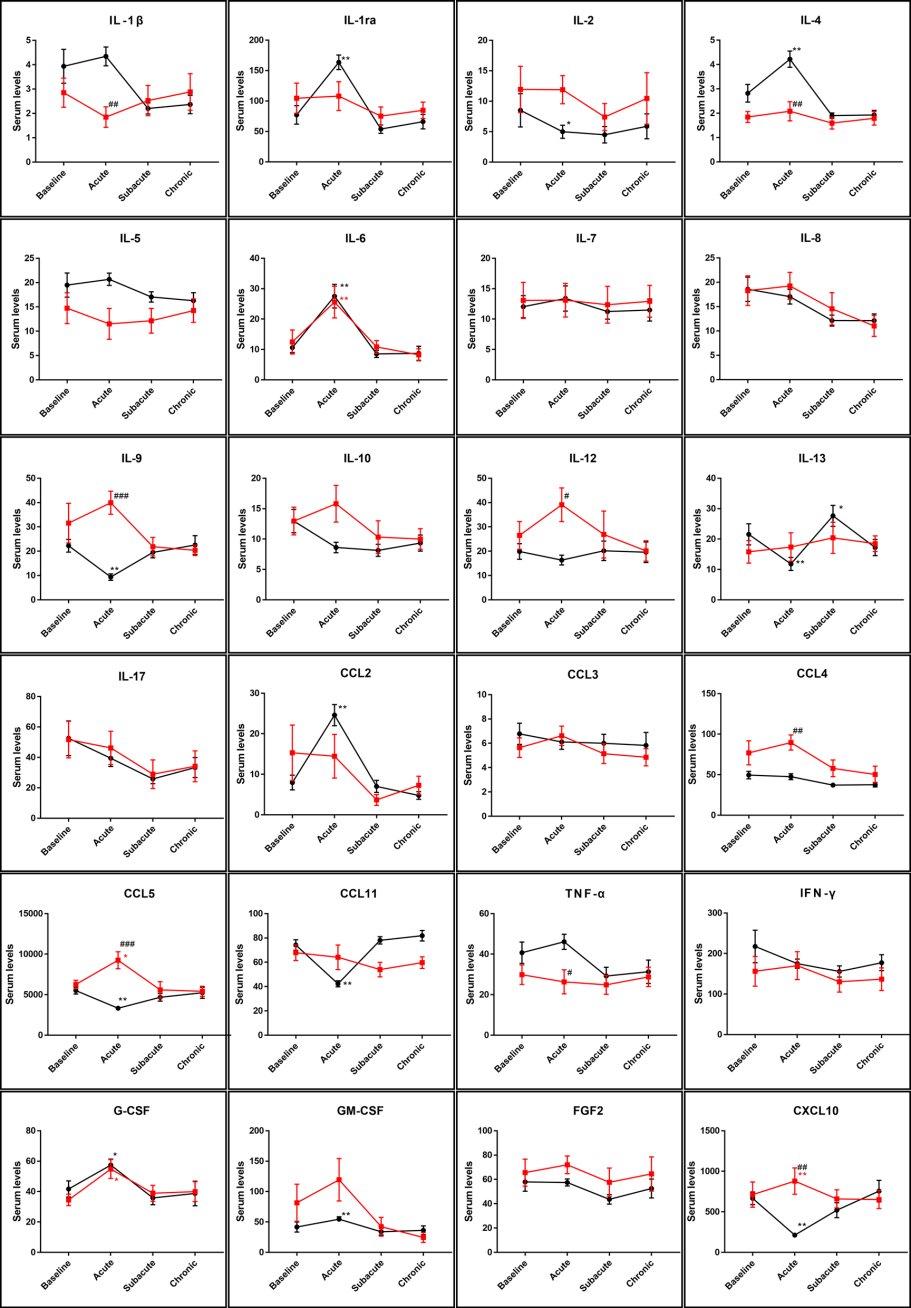
Changes in systemic cytokines in patients with or without cell sheet engraftment. Cytokines were measured before ESD, during acute phase, at subacute phase and in chronic phase after ESD. Black line represents the results from patients without cell sheet and red line represents the results from patients with cell sheet. Bars indicate average ± SEM. Dunnett test compared to baseline: *p < 0.05; **p < 0.01; Welch t test between patients with and without cell sheet: ^#^p < 0.05; ^##^p < 0.01; ^###^p < 0.001.

Next, we investigated the changes in cytokine levels after ESD in patients with or without cell sheet engraftment. The levels of IL-1ra, IL-4, IL-6, CCL2, G-CSF, and GM-CSF were significantly higher, whereas IL-9, IL-13, CCL5, CCL11, and CXCL10 levels were significantly lower in patients without cell sheet engraftment in the acute phase. Most of these changes returned close to baseline levels at the subacute phase (Fig. 2). Cell sheet engraftment significantly influenced IL-1β, IL-4, IL-9, IL-12, CCL4, CCL5, TNF-α, and CXCL10 levels (Fig. 2).

**Figure 3:**
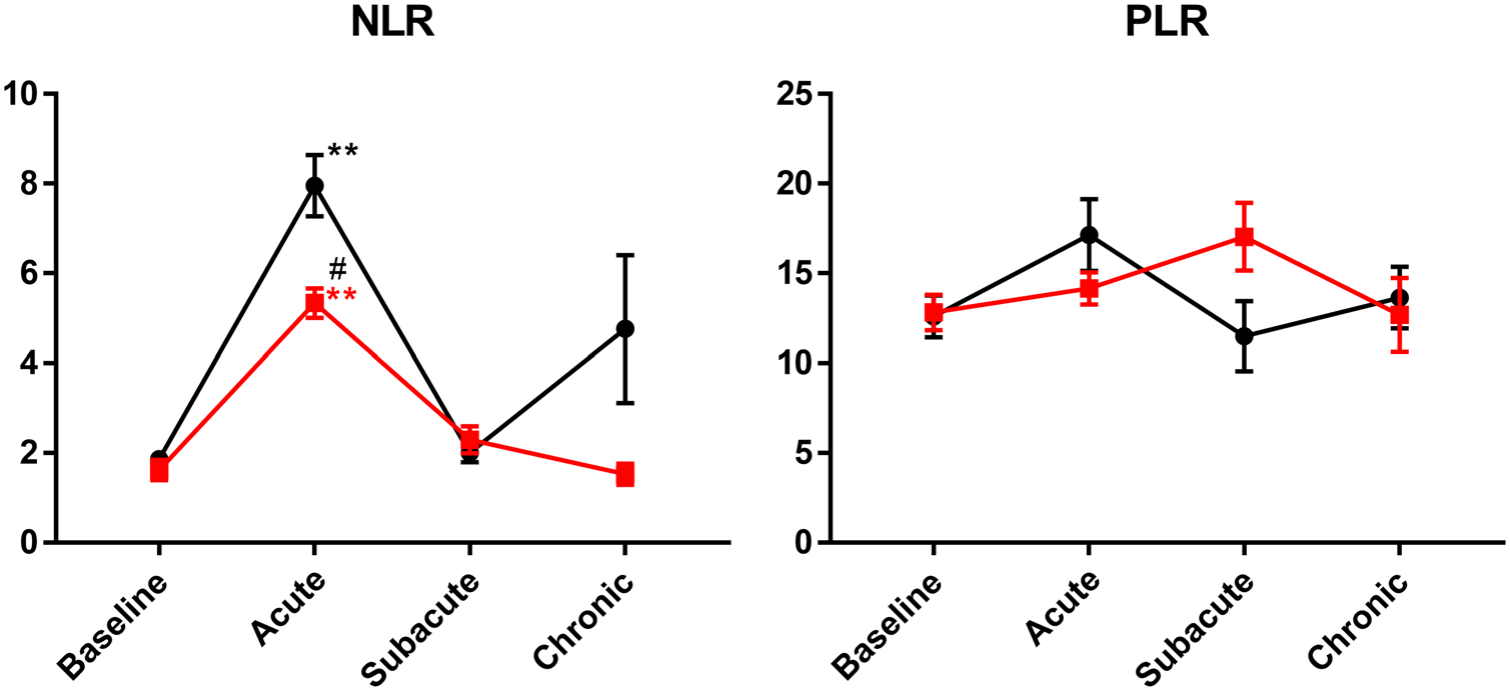
Changes in NLR and PLR in patient with or without cell sheet engraftment. NLR and PLR were measured before ESD, during acute phase, at subacute phase and in chronic phase after ESD. Black line represents the results from patients without cell sheet and red line represents the results from patients with cell sheet. Bars indicate average ± SEM. Dunnett test compared to baseline: **p < 0.01; Welch t test between patients with and without cell sheet: ^#^p < 0.05. *NLR* neutrophil-to-lymphocyte ratio, *PLR* platelet-to-lymphocyte ratio, *ESD* endoscopic submucosal dissection.

We further investigated the changes in the NLR and PLR in patients with oesophageal squamous cell carcinoma. The NLR increased significantly in the acute phase in both groups, and retuned close to baseline levels at the subacute phase. No significant change was observed in the PLR over time (Fig. 3).

## Discussion

In the present study, we comprehensively investigated systemic cytokine levels in patients with oesophageal squamous cell carcinoma, as well as the influence of ESD on systemic cytokine levels. Various pro-inflammatory cytokine concentrations were elevated in patients with oesophageal squamous cell carcinoma, and ESD affected the levels of some cytokines in the acute phase, followed by recursion in the chronic phase. In addition, we described the impact of autologous cell sheet engraftment on cytokine concentrations. To our knowledge, this is the first study to reveal the changes in systemic cytokine levels over time after ESD and the effect of cell sheets on cytokine expression.

Increasing evidence has suggested an association between TME and oesophageal cancer development, progression, and metastasis. Cancer cells interact with TME through various cytokines. Most cytokines present in the TME in oesophageal cancer have pro-inflammatory effects and promote cancer proliferation^9^. In the present study, we observed that serum cytokines, including IL-5, IL-6, IL-7, IL-9, IL-10, IL-13, IL-17, CCL2, CCL4, CCL5, CCL11, IFN-γ, G-CSF, GM-CSF, FGF2, and CXCL10 were significantly higher in patients with oesophageal squamous cell carcinoma. In contrast, cytokines involved in tumour suppression such as IL-1ra, IL-2, and IL-12 were also elevated. Some of the changes observed in our study were compatible with those of previous reports^10–12^, whereas the other changes were contradictory, possibly due to the histological type of the cancer^13^. Since patients with squamous cell carcinoma were enrolled in our study, smoking and alcohol consumption might have influenced the circulating levels of cytokines. Several reports have investigated the relation between smoking and circulating inflammatory cytokines and found that smoking increases the circulating levels of IL-6, CRP, and CCL11, but decreases the levels of IL-1ra and IL-1β^15,16^. Similarly, inflammatory markers including IL-6 and CRP were elevated in heavy drinkers. In contrast, the association between alcohol consumption and inflammatory cytokines showed U-shape or J-shape^17^. Behavioural factors are known to influence cytokine levels, and although these factors were not analysed in this study, increased inflammatory cytokines especially the pro-inflammatory ones, might reflect the patient behaviour.

Considering that inflammation causes oesophageal strictures after ESD and that cytokine levels are high at baseline in patients with oesophageal cancer, the impact of ESD on cytokine levels is a concern. IL-1β, IL-6, and TNF-α are the major pro-inflammatory cytokines related to post-ESD strictures^14^. Although we observed a significant increase in IL-6 levels in the acute phase after ESD, it returned to baseline levels in the subacute and chronic phases. The concentrations of IL-1β and TNF-α did not increase after ESD in the acute phase, but tended to decrease in the subacute phase. We also showed that the expression of these major pro-inflammatory cytokines was significantly suppressed by cell sheet engraftment. In particular, IL-1β and TNF-α levels in patients who received cell sheet transplantation were significantly lower during the acute phase. Because the epithelialization process progresses one week after ESD and excessive inflammation causes oesophageal stricture^8^, suppression of pro-inflammatory cytokines in the acute or subacute phase would have a favourable effect on the protection of the post-ESD stricture. In the present study, we did not observe significant difference in the incidence rate of post-ESD stricture. Muscular layer damage during the ESD procedure and the longitudinal mucosal defect length were reported to influence the post-ESD stenosis^18^. Although we could not investigate these factors in this study, the relatively high circumference in the cell sheet group might have influenced the incidence rate of post-ESD stricture. Another factor that potentially influenced the circulating cytokine levels was the medication, particularly the proton pump inhibitors (PPIs). PPIs reduced the inflammatory cytokines in patients with non-erosive reflux disease^19^. Although no previous reports have investigated the effect of PPIs in patients who have undergone oesophageal ESD, all the patients in our study were treated with PPIs after ESD. Therefore, the possible bias due to such medication was not a concern in this study.

In the present study, we showed that the NLR increased in the acute phase after ESD. The NLR is reported to be a marker of systemic inflammation, tumour stage, and histological type of gastrointestinal carcinoma^20–22^. WBC and neutrophils are regulated by TNF-α, IL-1β, and IL-6. Therefore, elevation of the NLR after ESD is the result of these pro-inflammatory cytokines. Leukocytes are a major source of chemokines. A close association between NLR and inflammatory cytokines has been reported, and the systemic inflammation evaluated by NLR reflects the changes in inflammatory cytokines in patients with oesophageal cancer^23^.

There are some limitations to our study. Although we showed the comprehensive fluctuation of systemic cytokines involved in oesophageal cancer and ESD, the precise interaction between each cytokine has not been investigated. Further research is needed to elucidate the complexity of the cytokine crosstalk. Patients receiving cell sheet transplantation were neither treated with local nor systemic steroids. Although this is a confounding factor for evaluating the impact of cell sheet engraftment on cytokine concentrations, considering the similar incidence ratio of post-ESD strictures, cell sheet engraftment potentially suppressed the excess inflammatory response after ESD.

In conclusion, we revealed distinct cytokine dynamics after ESD in patients with oesophageal cancer. In addition, the impact of autologous cell sheet engraftment on cytokine fluctuation was demonstrated. This study can accelerate research on the TME and therapeutic strategies for oesophageal cancer.

## Methods

### Study population

This study included 28 patients with oesophageal squamous cell carcinoma who underwent ESD with more than half of the circumstance resection between January 2013 and October 2014. Among them, 10 patients underwent cell sheet transplantation. ESD, autologous blood sampling, oral mucosal tissue harvesting, and engraftment of cell sheets were performed as previously described^8^. This study was conducted in accordance with the Declaration of Helsinki and was approved by the Ethics Committee of Nagasaki University (approval number: 09062626). Written informed consent was obtained from all the patients. In terms of cell sheet transplantation, which may include stem cells, this study was conducted in accordance with the guidelines on clinical research using human stem cells set by the Ministry of Health, Labour and Welfare of Japan.

### Cytokine assay

Blood samples were collected before ESD, during the acute phase (1–3 days after ESD), at the subacute phase (1 week after ESD), and in the chronic phase (1–2 months after ESD). In addition, blood from 10 healthy controls (5 women and 5 men, 37–54 years of age) were also quantified for the assay. Collected serum was stored at − 20 °C until quantification. The measured cytokines included IL-1β, IL-1ra, IL-2, IL-4, IL-5, IL-6, IL-7, IL-8, IL-9, IL-10, IL-12, IL-13, IL-17, CCL2, CCL3, CCL4, CCL5, CCL11, TNF-α, IFN-γ, G-CSF, GM-CSF, FGF2, and CXCL 10. Quantification was performed using the Bio–Plex Pro™ Human Cytokine 27-plex Assay kit (M500KCAF0Y, Bio–Rad Co., Hercules, CA, USA) and an automatic immunoassay analyser (Bio-Plex 200 System, Bio-Rad Laboratories, Inc.) according to the manufacturer’s protocol. Complete blood count and C-reactive protein (CRP) levels were also measured. The NLR was calculated as the ratio of neutrophil count to lymphocyte count, and PLR was calculated as the ratio of platelet count to lymphocyte count.

### Statistical analysis

Continuous variables are described as mean ± SD. The differences between two groups were analysed using the Welch t-test for continuous variables and the chi-square test for categorical variables. Differences in cytokine levels over time were analysed using two-way analysis of variance. If a significant difference was observed, Dunnett’s test was performed as a post hoc analysis. Differences between patients with or without cell sheet engraftment at each time point were analysed by Welch t-test with Bonferroni adjustment. A two-tailed p value of less than 0.05, was considered statistically significant. Statistical tests were performed using GraphPad Prism version (7.0. for Windows, GraphPad Software, San Diego, CA, USA) and StatFlex (6.0, for Windows, Artec, Osaka, Japan).

## Data Availability

The datasets of this study are available from the corresponding author on reasonable request.
